# MAP-Elites Enables Powerful Stepping Stones and Diversity for Modular Robotics

**DOI:** 10.3389/frobt.2021.639173

**Published:** 2021-04-28

**Authors:** Jørgen Nordmoen, Frank Veenstra, Kai Olav Ellefsen, Kyrre Glette

**Affiliations:** ^1^ Department of Informatics, University of Oslo, Oslo, Norway; ^2^ RITMO, University of Oslo, Oslo, Norway

**Keywords:** evolutionary robotics, modular robotics, morphology evolution, stepping stones, diversity, multiple environments, quality diversity, MAP-elites

## Abstract

In modular robotics modules can be reconfigured to change the morphology of the robot, making it able to adapt to specific tasks. However, optimizing both the body and control of such robots is a difficult challenge due to the intricate relationship between fine-tuning control and morphological changes that can invalidate such optimizations. These challenges can trap many optimization algorithms in local optima, halting progress towards better solutions. To solve this challenge we compare three different Evolutionary Algorithms on their capacity to optimize high performing and diverse morphologies and controllers in modular robotics. We compare two objective-based search algorithms, with and without a diversity promoting objective, with a Quality Diversity algorithm—MAP-Elites. The results show that MAP-Elites is capable of evolving the highest performing solutions in addition to generating the largest morphological diversity. Further, MAP-Elites is superior at regaining performance when transferring the population to new and more difficult environments. By analyzing genealogical ancestry we show that MAP-Elites produces more diverse and higher performing stepping stones than the two other objective-based search algorithms. The experiments transitioning the populations to new environments show the utility of morphological diversity, while the analysis of stepping stones show a strong correlation between diversity of ancestry and maximum performance on the locomotion task. Together, these results demonstrate the suitability of MAP-elites for the challenging task of morphology-control search for modular robots, and shed light on the algorithm’s capability of generating stepping stones for reaching high-performing solutions.

## 1 Introduction

Contemporary research in robotics commonly investigates how to adapt the controllers of robots when exposed to damage or changing environments. These studies usually consider robots with a fixed morphology. In modular robotics, morphological adaptation is achieved through the reconfiguration of modules (Yim et al., 2007). With this approach, different morphological configurations can accommodate various tasks and environments (White et al., 2005). However, the possible combinations of modules and control strategies are vast, giving rise to a nontrivial design challenge.

The field of Evolutionary Robotics (ER) approaches this challenge by applying Evolutionary Algorithms (EAs) to design and adapt both control and morphology of robots. EAs have been successfully applied to modular robot design and control (Hornby et al., 2003; Marbach and Ijspeert, 2004; Faíña et al., 2013) although they are prone to premature convergence (Cheney et al., 2016). Premature convergence is the phenomenon of having most, or all, solutions in the population converge to local optima, and the prospect of escaping these are difficult without sufficient diversity in the population (Hornby, 2006). This challenge is compounded when evolving *modular* robots due to the connection between controller optimization and morphology. In one relevant study, Faíña et al. (2013) observe a high degree of deceptiveness in the search landscape when evolving modular robots, leading standard EAs to underperform. In addition, with the available variation operators, such as adding a module, one may easily invalidate the current control strategy (Cheney et al., 2016).

Overcoming challenges in modular robotics require optimization algorithms that can evolve high performing solutions while retaining morphological diversity to avoid premature convergence in the resulting deceptive fitness landscapes. While little research exists so far on this challenge in the context of modular robotics, proposed approaches include a custom constructive approach (Faíña et al., 2013), morphological protection mechanisms (Cheney et al., 2018), or introducing a controller learning phase for new morphologies (Jelisavcic et al., 2019). These techniques essentially allow the morphology and control to change on different time scales.

Recent advances in ER are based on promoting phenotypic diversity in the search process. One simple but powerful way to achieve this is by making phenotypic difference in the current population an additional objective to maximize (Mouret and Doncieux, 2009). This multi-objective approach utilizes traditional Multi-Objective Evolutionary Algorithms (MOEAs) where one objective is the traditional performance value and another objective is added to represent the diversity of solutions (Mouret, 2011). Quality Diversity (QD) is an emerging paradigm within the field of EAs (Pugh et al., 2016). This class of algorithms go beyond the singular focus on maximizing one or more objectives and instead actively construct a repertoire of phenotypically unique and high-performing solutions (Cully and Demiris, 2017).

QD algorithms differentiate solutions based on phenotypic properties, also called behavioral descriptors, which dictate the inclusion into an archive. The most popular variants of QD algorithms are Novelty Search with Local Competition (NSLC) (Lehman and Stanley, 2011), which uses an unstructured archive, and Multi-dimensional Archive of Phenotypic Elites (MAPElites) (Mouret and Clune, 2015), which uses a structured archive: an N-dimensional grid spanning the behavioral descriptor space. Focusing on novelty instead of fitness alone has shown to find solutions in deceptive fitness landscapes (Lehman and Stanley, 2008), and QD algorithms have successfully been applied to evolve diversity of virtual creatures (Lehman and Stanley, 2011). In Miras et al. (2018a), a morphological novelty measure was included in a composite fitness function when evolving modular robots. However, the study focused on the diversity of the resulting morphologies, and locomotion performance was negatively affected compared to pure performance-based fitness function. Consequently, the application of a complete QD approach to tackle the above-mentioned challenges of evolving morphology and control for modular robots warrants exploration.

One potential reason for the efficacy of QD algorithms is the notion that QD algorithms are better at promoting and exploiting stepping stones (Mouret and Clune, 2015). In the context of EAs, we can describe a stepping stone, in its most basic form, as an *intermediate step to a final solution* (Mouret, 2020). In that way, a stepping stone does not need to have any other quality apart from being in the genealogical ancestry of the concluding solution. However, literature suggests that stepping stones to a complex and high-performing solution may consist of a range of very different solutions, seemingly unrelated to the objective (Secretan et al., 2011; Woolley and Stanley, 2011; Stanley and Lehman, 2015). In Mouret and Clune (2015) the authors propose that MAP-Elites is better at finding high performing solutions because the search algorithm is better at promoting diverse stepping stones. Another comparison of QD algorithms and objective-based search for the generation of stepping stones can be found in Gaier et al. (2019). Here the authors argue that due to the ability of MAP-Elites to protect poor, but ‘novel’ solutions, that can later be built upon to become good solutions, it is able to overcome premature convergence experienced with the objective-based approach. This suggests that analysing the potential of QD algorithms for generating stepping stones could be a way to increase our knowledge about this class of search algorithms and help explain the difference between QD algorithms and objective-based search methods.

Building on our initial study in Nordmoen et al. (2020b), this paper compares three EAs on their ability to evolve high performing and morphologically diverse modular robots. We utilize two objective-based search algorithms, one without a diversity objective and one with a diversity objective, and the QD algorithm MAP-Elites (Mouret and Clune, 2015) to illuminate the difference between these two paradigms as applied to the modular robotics domain. Our goal is to understand how the morphological difference evolved with these three search algorithms affect the task, when the environment changes and different morphological needs arise. Furthermore, to understand the algorithmic differences, we present an analysis of the genealogical ancestry of the evolved populations to shed light on the hypothesis that QD algorithms perform better due to a difference in how stepping stones are generated and utilized. To achieve the stated goals we created a new modular robotics framework Robotics, Evolution and Modularity (REM) which is used to simulate and evolve the modular robots for this paper.

The contributions of our paper are three-fold: First we demonstrate that MAP-Elites is well suited for the difficult task of evolving both morphology and control in modular robotics. By extending our previous results, we show that differing the selection pressure can have a large impact on maximum fitness obtained for this QD algorithm. We expand on the performance results by transitioning the populations of the three search algorithms between two different environments showing that as environmental complexity grows, the necessity for morphological diversity increases. Secondly, we present a way of analysing the genealogical ancestry of all three algorithmic approaches to better understand how properties of the ancestry can help explain the differences in maximum fitness. By looking at the statistical properties of the ancestry we gain the ability to generalize over all experimental runs which increases the confidence in our results. Finally, in addition to the two previous contributions, we release a new framework for evolving modular robotics, the REM framework, which leverages OpenAI Gym and PyBullet to achieve fast and easy to extend simulations, opening modular robotics up to a wider machine learning audience.

### 1.1 Related Work

#### 1.1.1 Modular Robotics

Evolving body and control for artificial creatures have a long history in the field of Artificial Life (Sims, 1994). Modular robotics is distinguished from these virtual creatures by comprising the morphology of re-usable homogeneous or heterogeneous building blocks, called modules (Stoy et al., 2010; Moubarak and Ben-Tzvi, 2012). This is in contrast to virtual creatures where individual body parts can evolve to have any shape and size. By using these building blocks, modular robotics provide a way to effectively transition from simulation to reality as modules can be fabricated individually and then combined based on designs optimized in simulation (Stoy, 2006). By designing modules in such a way as to make them easy to build in the real world, modular robotics offers a great deal of freedom for optimization in simulation since simulated robots can easily be put together from pre-built parts and transitioned to the real world for performance verification (Moreno et al., 2017). Through reusing modules and recent advances for potentially auto-assembling modular robots (Brodbeck et al., 2015; Moreno et al., 2018; Hale et al., 2019), this approach can become more feasible since the robot does not need to be constructed from the ground up.

One challenge in modular robotics is the interconnected relationship between control and morphology (Lipson and Pollack, 2000; Cheney et al., 2016). To overcome this challenge many different approaches such as generative encodings (Hornby et al., 2003; Veenstra et al., 2017) and different control architectures (Marbach and Ijspeert, 2005; Haasdijk et al., 2010) have been applied.

#### 1.1.2 Quality Diversity

QD algorithms emerged from the realization that optimization through promoting phenotypic diversity can yield high performing solutions and, more importantly, can be better suited to exploring the whole problem space (Lehman and Stanley, 2008). Through actively searching for phenotypic diversity, QD algorithms traverse the search space without constraining the search to only finding better-fit solutions (Pugh et al., 2016). This separates QD algorithms from traditional MOEAs since Pareto dominated solutions can be kept as long as their phenotypic expression is sufficiently different from other solutions in the population (Mouret and Clune, 2015). An interesting property of QD algorithms is the capability to produce a repertoire of different solutions for the same problem (Cully and Demiris, 2017). The repertoire can be exploited, either at design time (Gaier et al., 2017) or during operation (Cully et al., 2015), to select different solutions depending on the circumstances of the situation.

Although QD algorithms have been applied to the evolution of artificial creatures (Lehman and Stanley, 2011) and morphological descriptors have been used to evolve robots (Samuelsen and Glette, 2014; Samuelsen and Glette, 2015) few examples exist applying the QD paradigm to modular robotics. A related area of inspiration is voxel-based soft robotics (Hiller and Lipson, 2012). Several works have explored soft robot design with QD algorithms such as Methenitis et al. (2015) which first applied novelty search, Gravina et al. (2018) which combines novelty- and surprise search and Gravina et al. (2019) which compares different forms of diversity with MAP-Elites in the soft robotics domain.

## 2 Materials and Methods

### 2.1 Robotics, Evolution and Modularity Framework

For our experiments we created a new simulation framework based on PyBullet (Coumans and Bai, 2016) and OpenAI Gym (Brockman et al., 2016), called Robotics, Evolution and Modularity (REM).^1^ PyBullet is the Python interface to the Bullet (Coumans, 2015) physics simulator and OpenAI Gym is a framework to standardize simulations, initially within the reinforcement learning domain, that prescribes a few necessary functions that together create and run a simulation. OpenAI Gym makes it easy to reproduce setups from different experiments through exposing multiple environments through a common interface. Through using OpenAI gym we thereby make our framework more accessible, especially for those already familiar with OpenAI gym. By building the framework on PyBullet the orchestration code can be programmed in Python while Bullet itself is written and optimized in ‘*C*’.

**FIGURE 1:**
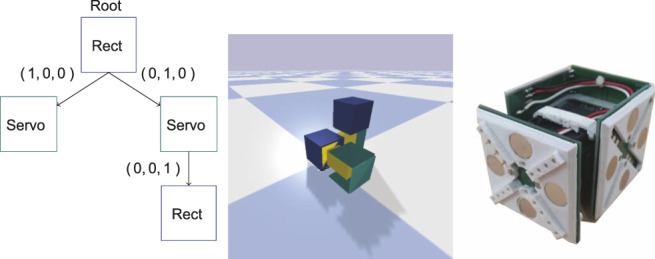
On the left the tree representation of the encoding is shown. The nodes in the graph represent modules and edges represent connections between modules. Each module has a certain number of possible connections where the triple (*X*, *Y*, *Z*) denotes where the connection is, relative to the parent, in 3D. Note that modules have additional properties, such as *rotation*, which is not shown in this illustration. In the middle the simulated modular robot corresponding to the encoding is shown in the REM framework. In the center is the root node, illustrated as a blue box. The root node has two direct connections, the two servo modules: one connected along its Y-axis and one connected along the X-axis. The servo module connected along the Y-axis has one rectangle module attached along its Z-axis. The rectangular modules have five connection sites to which other modules can be connected, corresponding to the surfaces exposed on the root module, and one which it can use to connect to others—which is the site underneath the root module. The joint modules have three connection sites which correspond to the exposed surfaces on the green bracket. The bottom of the joint module is used as a connection to other modules. On the right the real-world joint module is shown.

The modules supported by REM are based on the EMeRGE (Moreno et al., 2017) modules and have real-world properties for size, weight and joint forces. At the time of publication, the REM framework supports two different module types, one movable joint module which is based on Dynamixel AX-18—shown on the right in Figure 1, and a non-movable module with the same dimensions as the joint module. The connections between modules are based on magnets which makes the real-world modules easy to assemble and disassemble. Unfortunately, Bullet does not support connections that can break at a certain force threshold and so this is not supported yet. Simulation is default performed at 240 Hz, when graphical interface is not enabled, to give a high degree of accuracy for simulation.

To support different genome encodings the REM framework utilizes a tree-based acyclic graph representation for phenotypes which describes the morphology to instantiate. This allows for both direct and indirect genome encodings as long as they unpack into the graph expected by REM.

### 2.2 Encoding and Control System

The morphological encoding employed in the experiments is a tree-based direct encoding similar to Faíña et al. (2013), illustrated in Figure 1. The encoding allows for any directed acyclic graph of modules to be represented, where each node in the graph represents a module and each edge is a connection between two modules. The encoding corresponds one-to-one with the phenotype encoding in the REM framework. For the experiments carried out in this article two different modules were utilized, one non-movable rectangular module supporting five child modules and one servo module capable of moving one side back-and-forth and supporting three child modules (Moreno et al., 2017). Each morphology starts with a single rectangular module as its root. To randomly initialize the morphology, a random size is selected between one and η. Modules are then added to the tree at random locations until the size of the morphology equals the selected size.

The morphological encoding supports mutation- and crossover-operators. When mutating the morphology three possibilities exist: 1) *Add a random module.* The tree is traversed and each available connection point is added as a possibility. A connection point is randomly selected along with a randomly selected module type before being inserted into the tree. 2) *Remove a module.* The tree is traversed adding all modules, except the root, into a list of candidates to remove. A module is randomly selected from the candidates before being removed along with any existing children. 3) *Rotate a module.* The two modules in use both support rotation around its connection axis and mutation will randomly select a new orientation in 90° increments. Note that *only* one of the three possible changes can occur per morphological mutation. These mutations cannot alter the type of a module.

For crossover, a branch exchange is implemented. For both parent morphologies the tree is traversed adding all modules, except the root, to a list of candidates. A random candidate is selected from both morphologies before being exchanged. The candidate module, including its children, from the first morphology, is inserted into the place of the candidate from the second morphology and vice versa.

Lastly, the morphology is limited to a maximum size, η=20, and a maximum depth δ=4, so that additional modules are not realized in the simulator. This limit ensures that morphologies do not grow unbounded and are feasible to simulate.

The control systems of the joint modules are based on a decentralized wave pattern generator (Veenstra et al., 2017). Each joint module is initialized with a controller that is updated on each simulation tick to output desired angle of the joint, $\theta_{i}$, according to the following equation

$$\theta_{i}=\alpha_{i}\cdot \text{sin}\left(\omega_{i}t+\phi \right)+o_{i}$$ (1)

where $\alpha_{i}$ is the amplitude, *t* is the time since the controller was initialized, $\omega_{i}$ is the frequency, $\phi_{i}$ is the phase offset and *o*_*i*_ is the amplitude offset. The output of the controller, i.e., the maximum and minimum values of $\theta_{i}$, is limited so it does not exceed the ability of the real world module. The parameters and their allowable ranges are defined in Appendix 1.

The controllers are mutated using Gaussian noise, N(p,σ), where *p* is the individual parameter of the controller and *σ* is the magnitude of the noise. The magnitude, *σ*, is scaled for each parameter so that a global mutation rate can be used for the controller, the scaling is defined by the range of each parameter detailed in Appendix 1. To avoid mutating values outside their defined bounds we utilize the *bounce-back* restriction function (Nordmoen et al., 2020a), which restricts values to a boundary condition by reflecting the value back inside the boundary by the amount of over- or undershoot.

### 2.3 Evolutionary Algorithms

To better understand how QD algorithms are able to evolve both high performance and diverse solutions we will compare three different EAs on the task of evolving both control and morphology in modular robotics. The comparison will initially utilize a flat terrain environment before experimenting in more complex simulated environments. The fitness objective of the EAs is the straight-line distance traversed, between the initial starting point and final position of the robot, during the evaluation.

For the diversity preserving EAs, morphological properties will be used to distinguish solutions. Selecting which morphological properties to utilize is a challenging problem, and may have a significant impact on the properties of the search space (Samuelsen and Glette, 2014; Miras et al., 2018b). In this paper we define the number of non-movable and the number of movable joint modules as morphological properties that can be used as our diversity metric. These features are relatively simple but combined they will relate to properties like size and agility of the robot morphologies, and should allow for a range of different morphological strategies, while being easy to use in conjunction with the applied search algorithms. By constraining our diversity metric to these two simple features we try to limit any added complexity that comes with adding more (and potentially more powerful) descriptors, and rather focus on the algorithmic approaches in these experiments.

The first EA is a single objective, (μ,λ) generational replacement strategy, based on Eiben and Smith (2003). The algorithm optimizes for fitness alone and is used as a baseline to compare the two other diversity preserving algorithms. The algorithm utilizes tournament selection between two solutions, based on fitness, for selection and incorporates elitism, preserving 10 of the best solutions from the previous generation. For the rest of this article we will refer to this algorithm as Single Objective Fitness Only (SOFO). Experiment parameters for this algorithm can be found in Table 1.

**TABLE 1:** Experiment parameters for the search algorithms.

**Parameter**	**Applied to**	**Value**
Evaluation time	All	20
Warm-up before start		2 s
Repetitions		30
Number of evaluations		100,000
Batch size		200
Probability of crossover		0.2
Probability of controller mutation		1.0
Initial population size	SOFO	200
	MOFD	
	QDSA	1000
Selection	SOFO	Tournament on objective(s)
	MOFD	
	QDSA	Tournament on *curiosity*
Probability of morphological mutation	SOFO	0.2
	MOFD	
	QDSA	0.4
Controller mutation magnitude (*σ*)	SOFO	0.01
	MOFD	
	QDSA	0.005

The first of the diversity preserving algorithms is the MOEA, Non-dominated Sorting Genetic Algorithm-II (NSGA-II) (Deb et al., 2002). This EA also represents the objective optimization perspective, however, since NSGA-II is capable of optimizing multiple objectives the algorithm can be used to simultaneously optimize for diversity. This approach of using diversity in addition to the main objective is well-studied and has been shown to be efficient in the domain of ER (Mouret and Doncieux, 2012). In our study, the diversity metrics used are based on morphological descriptors of the evolved robots and comprise the number of non-movable modules and the number of movable modules. For NSGA-II to optimize for diversity, the average difference between morphologies is used as an objective (Lehman and Stanley, 2011), according to the following equations:

$$diversity\left(x\right)=\frac{1}{\left|P_{n}\right|}\sum_{y\in P_{n}}distance\left(x,y\right)$$ (2)

$$distance\left(x,y\right)=1.0-e^{\left|\left(m_{x},j_{x}\right)-\left(m_{y},j_{y}\right)\right|}$$ (3)

where _*Pn*_ is the population, *x* and *y* are solutions in the population, _*mi*_ is the number of non-movable modules and _*ji*_ is the number of movable joint modules. Note that we treat each module type separately, which means the output of both equations is given in ${\mathbb{R}}^{2}$-which gives three objectives for NSGA-II to optimize: One diversity objective for each module type and one fitness objective. Note also that the distance equation is altered compared to some previous works (Lehman and Stanley, 2011; Samuelsen and Glette, 2014) to avoid convergence at the morphological extremities. By changing the distance function (Eq. 3) to using the natural exponential function all changes in morphology are weighted equally, which prevents large changes in morphology from dominating the diversity calculation during optimization. In effect, adding one or ten modules is weighted as equally diverse. For the rest of this article we will refer to this algorithm as Multi Objective Fitness and Diversity (MOFD). Experiment parameters for the algorithm can be found in Table 1.

The last EA used represents the QD paradigm and is the MAP-Elites algorithm (Mouret and Clune, 2015). Central to the MAP-Elites algorithm is the archive, or repertoire, which is utilized to store and select solutions. The archive is structured with cells of equal sizes that represent a specific combination of feature descriptors (Cully and Demiris, 2017). As with MOFD, we utilize morphological properties as feature descriptors. In contrast to MOEAs, MAP-Elites does not utilize multiple objectives, however, diversity is promoted through the archive by allowing multiple solutions to be differentiated by their feature descriptors. For the experiments carried out in this article, the archive consists of two dimensions where one axis represents the number of non-movable modules and the other axis represents the number of movable joint modules. The dimensions are scaled to the maximum size of a morphology and the cell at the origin represents the root module. For selection we utilize tournament selection based on the *curiosity* of solutions in the repertoire (Cully and Demiris, 2017). Curiosity is implemented by adding 1.0 to the curiosity score of a parent when a child is inserted into the repertoire and subtract 0.5 when a child fails to be inserted into the repertoire, which corresponds to the values suggested in Cully and Demiris (2017). For consistency, we will refer to this search algorithm as Quality Diversity with Structured Archive (QDSA). Parameters used for experiments for this algorithm can be found in Table 1.

**FIGURE 2:**
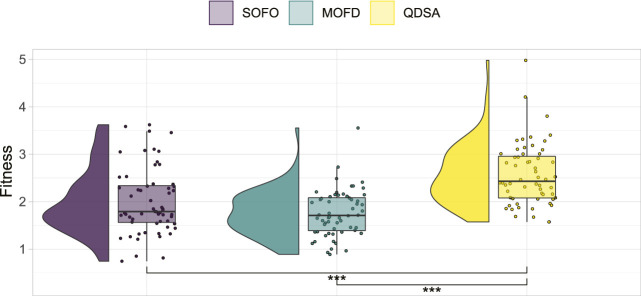
Summary of parameter optimization. All 60 runs for each search algorithm are shown together with the result of a Mann-Whitney *U* test which show statistically significant differences between QDSA and the two other search algorithms.

To ensure a balanced comparison, the mutation parameters of each algorithm were optimized in advance in a parameter sweep (parameters detailed in Appendix 1). Each parameter was tested twice for each EA and 100,000 evaluations were done for each set of combined parameters. The simulation time for each evaluation was limited to 20 s in these initial runs. In total 180 runs were conducted to ascertain the best parameters for each search algorithm. Based on these results, a linear model was constructed to predict fitness based on the interaction of the two parameters tested. The best parameter combination of each algorithm was chosen to be used in the remaining experiments of this paper. A summary of all the runs is shown in Figure 2, which shows that QDSA is on average slightly better than the two other search algorithms—regardless of parameter combination.

### 2.4 Objectives

**FIGURE 3:**
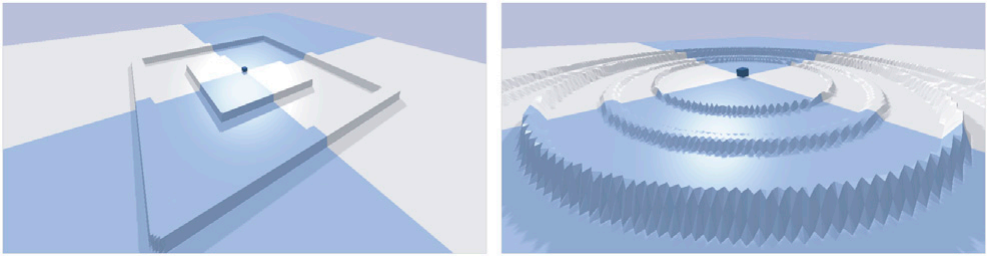
The more complex environments used to test transferred solutions. On the left the robots start on a raised platform until a ditch is created with a single wide wall. On the right the robots start on a flat terrain and several circular thin walls ripple outwards becoming taller and taller.

The environment where the robots must move in shape the search space of our experiments. With a flat terrain, an evolutionary run might lead to a smooth progression due to the absence of obstacles/deceptive traps. To see whether all the approaches perform the same when changing the environment, we use three different environments: A flat terrain, a raised platform with a single wall, and a circular terrain where circular walls ripple outwards (Figure 3).

## 3 Results

### 3.1 Performance and Diversity

**FIGURE 4:**
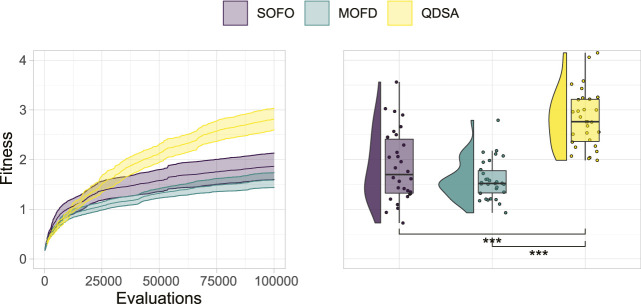
Fitness of the single best solution found in the population. On the left the mean is shown together with a 95% confidence interval over generational time. On the right, the best fitness after the last evaluation for all repetitions is shown. Statistically significant differences are marked on the right using a Mann-Whitney *U* test with Holm correction.

To begin analysing the performance of the three search algorithms, we will start by looking at the best fitness obtained by any single solution in the population. The best fitness is plotted in Figure 4, where on the left the fitness is shown over the number of performed evaluations, and on the right the single best individual found after the last evaluation is shown. A Mann-Whitney *U* test (Mann and Whitney, 1947) between the three distributions in the right plot of Figure 4, corrected for multiple comparison through Holm correction (Holm, 1979), shows that there is a significant difference between QDSA and the two other search algorithms. For locomotion, QDSA is able to find the best performing solution of the three search algorithms.

**FIGURE 5:**
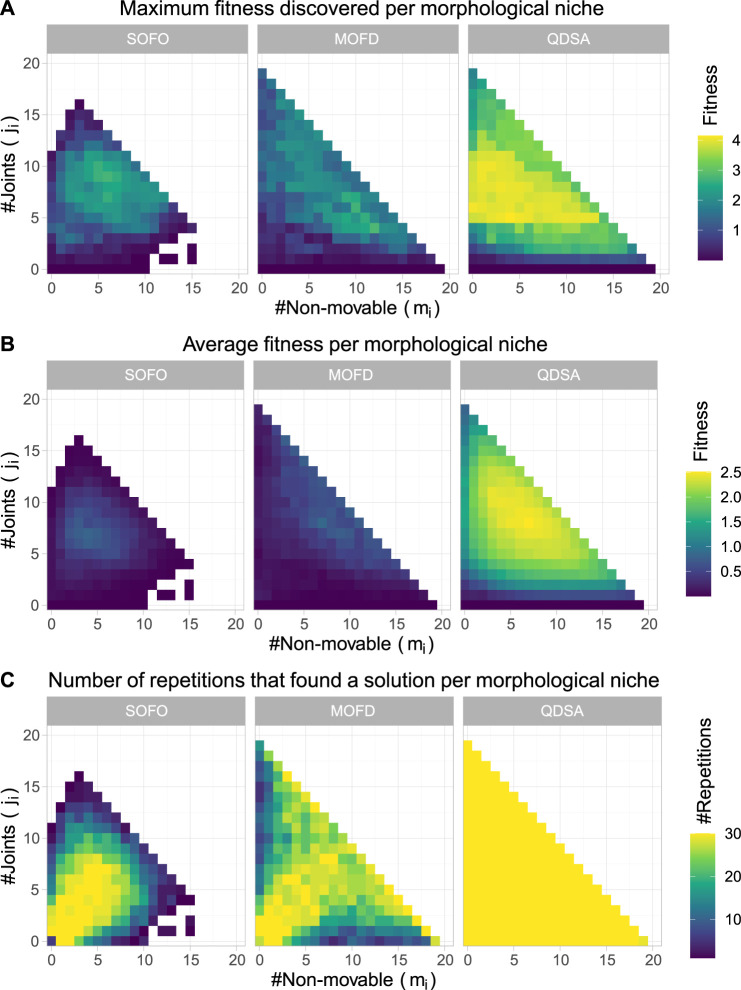
The figures show properties of the population for each search algorithm over all 30 runs. **(A)** shows maximum fitness of morphological niches, where the color represents fitness. **(B)** shows the average fitness, where for each niche the color is the average fitness calculated over the 30 runs. **(C)** shows the number of repetitions that found a solution for the given niche.

Since it is not only the fitness, or quality, of the solutions that we are interested in, it is informative to project the population of solutions into a repertoire using the morphological descriptors as axes. This projection gives an overview of the population as a whole and it is possible to visualize where in the morphological space the best solutions are found. The projection also enables us to visualize the quality-diversity trade-off, which shows that not all solutions, or morphologies, can obtain the same fitness. For the objective based search algorithms the projections are created by inserting solutions after each generation, accumulating solutions as the search progresses. In Figure 5 the top row shows the maximum fitness for each morphological niche, while the middle row shows the average fitness. The two figures illustrate the difference in diversity between the different search algorithms and shows how consistent the algorithms are at discovering solutions.

Although the middle row of Figure 5 shows diversity of the search algorithms, through the average fitness of morphologies, it is not able to show how proficient each algorithm is at finding diverse solutions, low performance may just indicate that the morphological niche cannot perform better. To alleviate this, the bottom row shows the number of experiments which found a solution for each morphological niche. From the figure it can be seen that QDSA and MOFD are more consistent in finding diverse solutions while the single objective SOFO is centered around a smaller cluster of morphologies. Although MOFD is able to find more diverse solutions than SOFO, only QDSA consistently finds solutions for all niches.

**FIGURE 6:**
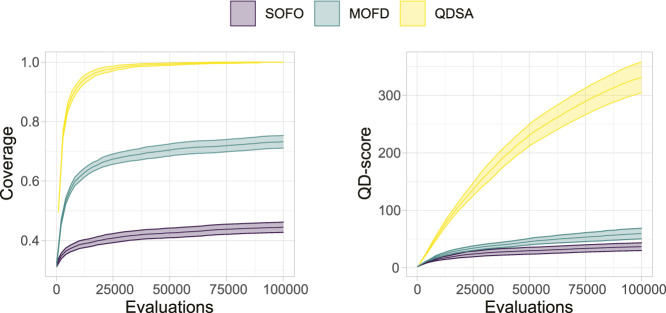
QD-Metrics. On the left the normalized coverage (Mouret and Clune, 2015), number of discovered morphological niches, is shown. Coverage has been normalized to the maximum number of niches found in any run for all three search algorithms. On the right the QD-score (Pugh et al., 2016), summation of fitness for all solutions in the population, is shown. Both plots show the mean and a 95% confidence interval.

To summarize the projections in Figure 5 we can utilize metrics suggested by Mouret and Clune (2015) and Pugh et al. (2016). Figure 6 shows the coverage and QD-score of the three search algorithms. Coverage counts the number of unique niches found in the population and is normalized to the maximum coverage found in any run of all algorithms. Coverage can be viewed as a summation of the data shown in the bottom row of Figure 5 and shows the evolved diversity of the search algorithms. QD-score is the sum of fitness of each solution in the population and is a good summation of the quality and diversity trade-off. QD-score gives a more balanced view than either *precision* or *reliability* since both of these metrics decrease as new low fitness solutions are added due to the lower average performance, which disadvantages search algorithms that generate diversity. A Mann-Whitney *U* test demonstrates that the differences between all three search algorithms, after the last evaluations, for both plots in Figure 6, are significant. The two graphs in Figure 6 show the complexity of comparing algorithms on the trade-off between quality and diversity, even though MOFD has a much higher coverage compared to SOFO the difference in QD-score is much lower due to SOFO having on average high fitness in the niches it occupies.

**FIGURE 7:**
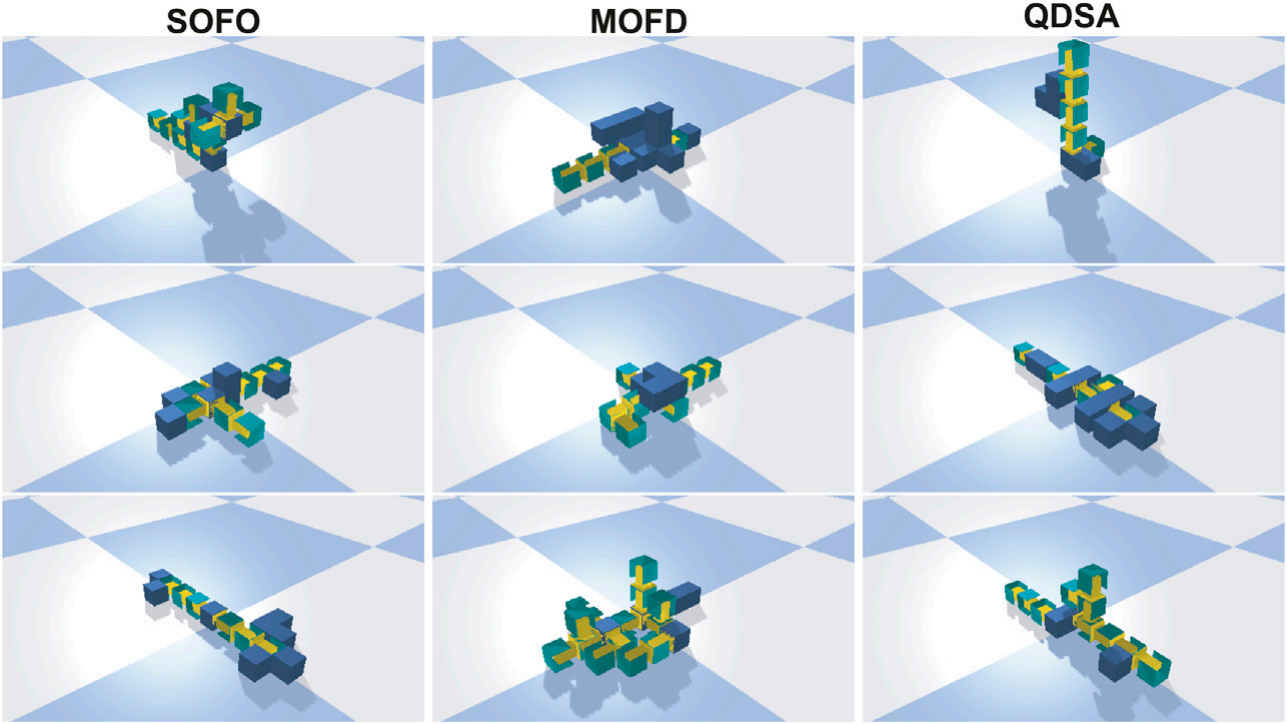
The best solution found in the three best runs of evolution for each search algorithm. Videos of these morphologies can be found on the supplementary material page.

To get an impression of the evolved morphologies, we selected the three best runs from each search algorithm and extracted the single best solution. The solutions are shown in Figure 7.

### 3.2 Transitioning to New Environments

**FIGURE 8:**
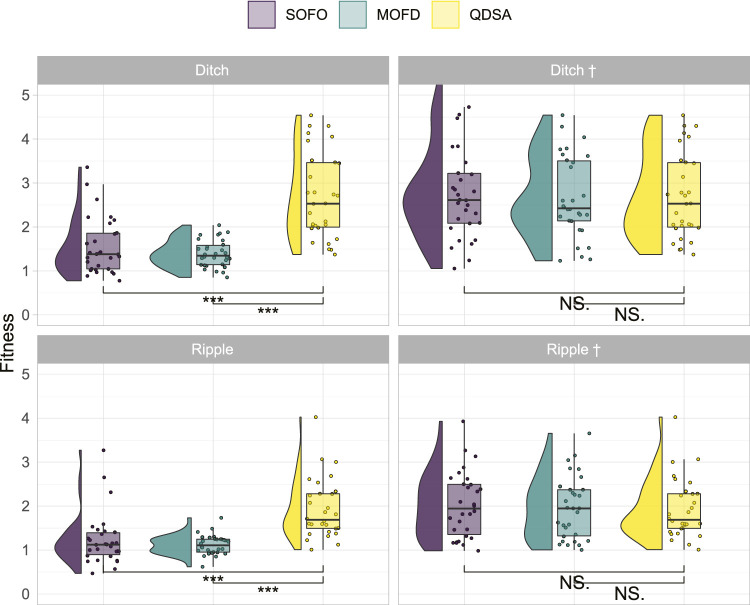
Fitness results after last evaluation for transitioning the population evolved in the default flat environment into two new environments. In the new environment evolution is continued from the initial seed population for 50,000 evaluations. Names with † signify that both SOFO and MOFD was initialized with the result of QDSA from the default flat environment.

To better understand the value of diversity in our modular robotics scenario we created two new environments, with different obstacle profiles as shown in Figure 3, to see if the difference in evolved morphologies would lead to differing results in more challenging environments. The hypothesis being that a more diverse population should transition better into a different environment since an already discovered morphology could potentially lead to good performance in the new environment. Said in another way, convergence to a few good solutions in one environment could lead to slow evolution in another environment if none of the converged solutions are able to solve the new environment. We tested this hypothesis by transitioning the final population in Figure 4 into two different environments and ‘continue’ evolution from the population evolved for the default environment. The results are shown in the left column of Figure 8. From the left column it can be seen that QDSA is able to obtain the best fitness in both new environments. The difference between SOFO and MOFD is not significantly different, however, it is interesting to note that they seem to have changed relative position compared to Figure 4, a change that could indicate that the diversity of MOFD is aiding in transitioning into a new environment. In addition to testing the result of each search algorithm we also tested if the population evolved in the default environment with QDSA could aid the other two algorithms. The population of QDSA was transitioned from the default environment into the two new environments, but instead of using as mentioned in Cully and Demiris (2017) the two other search algorithms were utilized to continue evolution. The results of continuing from the population of QDSA in the two new environments are shown in the right column of Figure 8. Here it can be seen that there are no significant differences between the three search algorithms.

**FIGURE 9:**
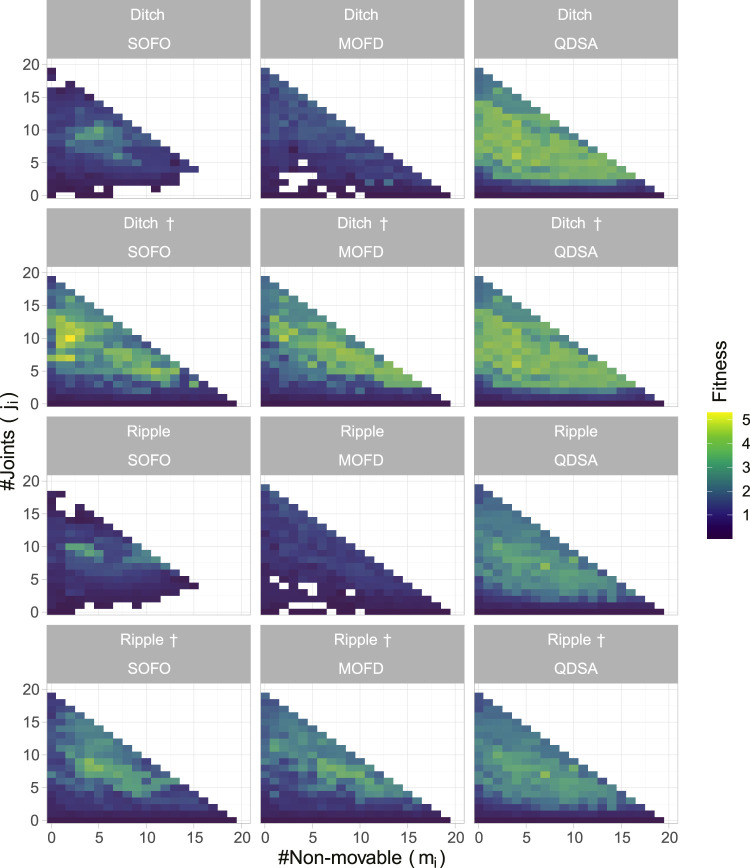
Population projection in the different environment settings. The projections show the best solution for each morphological descriptor over all runs after the last evaluation, where color represents fitness. Environment names with † signify that both SOFO and MOFD were initialized with the result of QDSA from the default flat environment.

To highlight the difference between the evolved populations in the different environmental settings, we projected the best-found solutions for the different morphological descriptions in Figure 9. This figure shows that both SOFO and MOFD are able to solve the more challenging environments when initialized with the result of QDSA. However, when started from their respective previous population from the flat environment they are not able to regain the same fitness as MOFD. Note that the figures show the cumulative best solution which accounts for the large difference in number of filled cells for the † environments.

### 3.3 Genealogical Analysis

**FIGURE 10:**
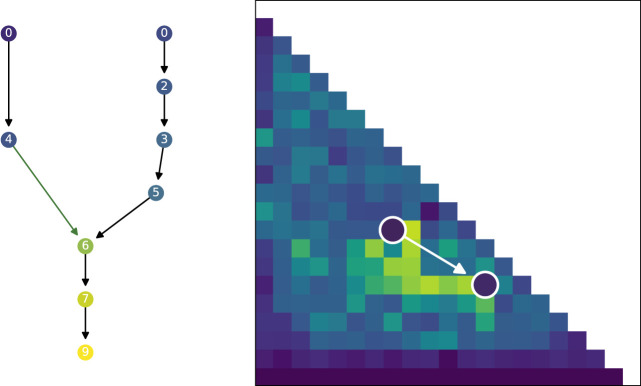
Visualized ancestry of a solution. On the left the circles represent solutions that are ancestors of the bottom most circle. The color of the circle represents the fitness of the solution. The number is the generation when the solution appeared in the population. Arrows indicate parenthood, where black arrows indicate that there is *no* morphological difference and green arrows indicate a morphological difference between parent and child. The two arrows joining to one new solution corresponds to a crossover operation while the other arrows correspond to mutation operations. On the right the tree is projected back into a repertoire and shows that the whole tree developed in just two different morphological descriptions, the white arrow indicates a change from parent to child in morphology.

Up until now the focus has been on the quality and diversity of the evolved populations, however, the previous graphs have not been able to show why the search algorithms evolve differently. We will therefore analyze the genealogical history of solutions to better understand how the solutions evolved and formed stepping stones. Figure 10 shows an illustration of the genealogical ancestry that we will analyze. The ancestry tree of a single solution is shown, as well as the ancestry’s projection into the morphological niches it occupies. The figures in this section are created by taking each solution in a given generation, extracting their genealogical ancestry—as shown in Figure 10—calculating various statistics on the whole ancestry and then collating the results of all the individual solutions in the considered generation.

**FIGURE 11:**
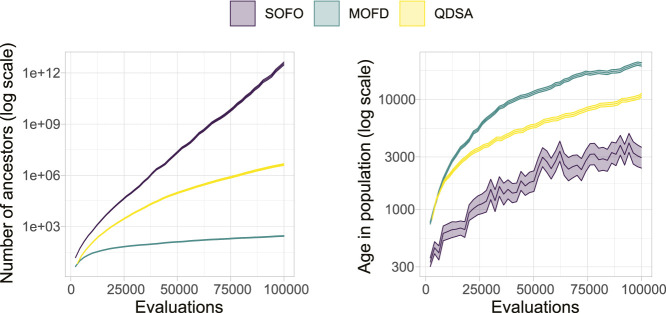
Genealogical ancestry attributes. On the left the average number of ancestors for each solution in the population is shown. On the right the age of each solution is shown, where age is number of evaluations since the solution first appeared in the population. The colored area represents the 95% confidence interval.

Figure 11 shows the number of ancestors and the age of solutions over generational time. The number of ancestors is simply the size of the ancestry, for the example in Figure 10 the number of ancestors would be 8, and gives an indication of how often solutions are replaced. Another way to look at this replacement is to measure the age of solutions. Age in all examples is measured as the number of evaluations performed by the algorithm since a solution appeared in the population. For QD this means that newly inserted solutions in the archive will have an age of 0 that could have replaced older less fit individuals in the same location of the archive. Figure 11 illustrates that the generational replacement EA creates a lot of new solutions, making the average age in the population very low, while the two other search algorithms tend to generate fewer new solutions and thus have a higher age.

**FIGURE 12:**
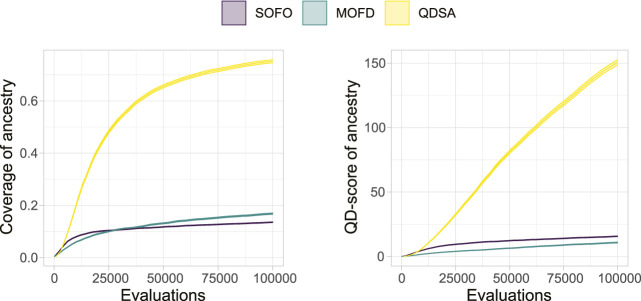
Quality Diversity metrics applied to the genealogical ancestry of each solution in the final population, after the ancestry is projected into a unique repertoire as illustrated in Figure 10. On the left, coverage is shown, which is the number of morphological niches occupied by the ancestry. On the right, the QD-score is shown, which is a summation of the fitness of the ancestry after the ancestry has been projected into a repertoire. The figure shows the mean and a 95% confidence interval.

As shown in Figure 10, projecting the ancestry into a repertoire of morphological niches can be a way to gain insight into how a solution evolved over time. In other words, the projection discards some information from the complete ancestry tree and focuses on changes in features, as defined by the feature descriptors. In our case, this corresponds to when morphological changes are introduced. A projection covering a large portion of the feature space could thus be interpreted as having a large amount of different stepping stones. In Figure 12 the quality-diversity metrics coverage and QD-score are applied to the *ancestry* of solutions in the population. This summary is different from the data in Figure 6 as this utilizes the genealogical ancestor tree and projects that into a unique repertoire for each individual solution in the population, before applying the two metrics. The difference being that for the genealogical ancestry each solution in the population is used to generate a unique repertoire consisting of only ancestors of the concluding solutions before QD metrics are applied to each of these ‘ancestor repertoires’. Figure 12 shows the coverage of ancestry (left), and on the QD-score (right). From the figures it can be seen that the solutions in QDSA have an ancestry which covers a larger fraction of the morphological search space. This is contrasted with MOFD which is able to obtain quite good coverage, as seen in Figure 6, while the ancestry of solutions tends to have a much lower morphological diversity. One way to interpret this is that solutions in QDSA tend to share more ancestry with morphologically different solutions compared to MOFD.

**FIGURE 13:**
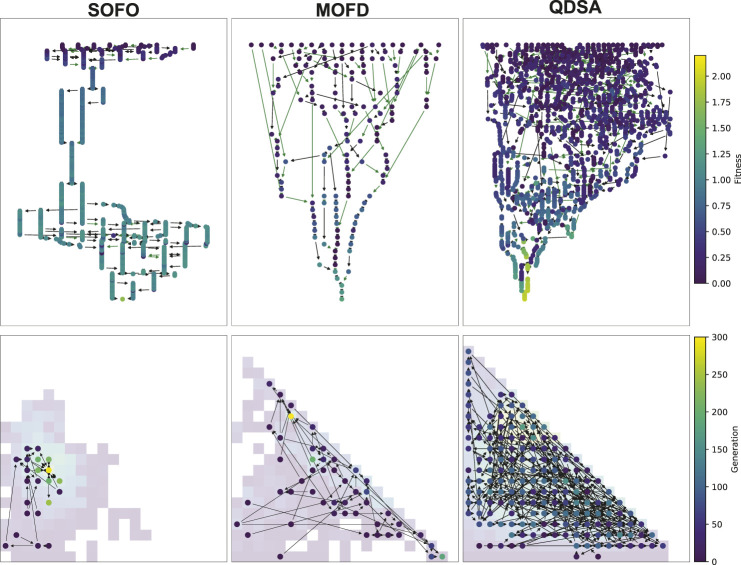
Visualized ancestry from a randomly selected seed. The top row shows the ancestry of the most fit solution for the algorithm and the bottom row shows the same ancestry projected down into a MAP-Elites repertoire, note that for SOFO and MOFD the repertoire shows the accumulated population throughout evolution. The color of the nodes in the top row corresponds to fitness while the color of the nodes in the bottom row shows the generation when the solution appeared in the search.

To illustrate these genealogical statistics we selected a random run and plotted the ancestry of the best solution found during evolution. Figure 13 shows the visualized ancestry which underscores the above statistics. From the figure we can see that SOFO and QDSA have the largest ancestry trees. However, when projected down into the repertoire we can see that SOFO covers only a small area of the morphological search space, while QDSA covers the largest area.

**FIGURE 14:**
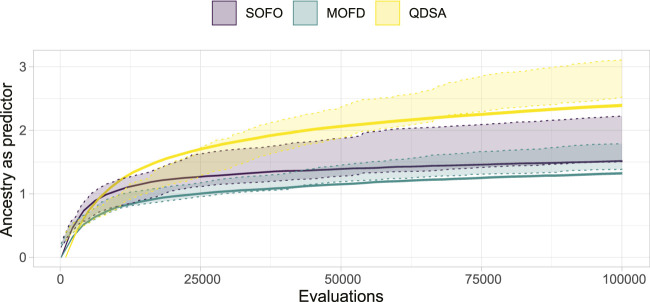
Using Quality Diversity metrics of ancestry to predict maximum fitness of each search algorithm. The dotted area represents the 95% confidence interval of maximum fitness, as shown in Figure 4, and the line represents the estimated fitness based on a linear model of Quality Diversity metrics of ancestry. The linear model achieves an *R* ^2^ of 0.9084.

To investigate a possible connection between the QD properties of the ancestry, as shown in Figure 12, and the performance of the algorithms, we created a linear model as an analysis tool. The linear model predicts maximum obtained fitness based on logarithmic coverage and QD-score, both from ancestry. The model fit the data with an *R* ^2^ of 0.9084, which indicates that the model fit the data quite well, coefficients can be found in Appendix 1.^2^ To verify if the model fit with the maximum fitness of Figure 4, we plotted the 95% confidence interval of the fitness, as shown in Figure 4, overlaid with the estimated fitness based on coverage and QD-score in Figure 14. From the figure it can be seen that the model is challenged by the larger difference in ancestry between the three search algorithms, Figure 12, compared to the lower difference in maximum fitness, Figure 4. The figure illustrates that the model, for a large part of the data, matches the obtained maximum fitness and thus could be an indication that these ancestry metrics are a good predictor of maximum fitness.

## 4 Discussion

The results for the locomotion task, in the default flat terrain, shows that QDSA produced the highest performing solutions for this problem (Figure 4). For the experiments in this paper we added a curiosity score that led to increased performance of QDSA within our computational budget. Since our previous work (Nordmoen et al., 2020b) showed no quantifiable difference between the three search algorithms, the addition of a curiosity score and selection on this score, corroborates the previous findings mentioned in Cully and Demiris (2017) of curiosity being a useful addition to MAP-Elites. From the performance results, it can also be seen that QDSA produces the most diverse populations going as far as filling out all the morphological niches defined and is the most consistent at finding high performing solutions (Figure 5).

With respect to morphological diversity we showed that explicitly promoting diversity through either objective- or QD-based search algorithms improved the assortment of morphologies. However, QDSA produces more diversity compared to MOFD, demonstrating the advantage of QD algorithms for this task. Coupled together, it is likely that the diversity produced in QDSA together with the selection pressure to improve in promising areas of the search landscape led to higher overall performance, underscoring the utility of diversity in EAs. The effect of this could potentially explain the slower rise in performance seen on in Figure 4 in contrast to the sharp growth of coverage in Figure 6. A potential explanation is that the search initially increases diversity, likely because it is easy to fill empty morphological niches, before being forced to improve performance of existing solutions.

When transitioning to different environments we showed that QDSA is able to gain significantly higher performance compared to the two other approaches. However, all approaches achieved equal fitness when seeded with the population of QDSA at the time of transition. The seeding also led to all three search algorithms finding good solutions in the same area of the search space (Figure 9). This shows the advantage of morphological diversity, as all three search algorithms are able to recover a large portion of fitness when initialized from a sufficiently diverse population. However, only the QDSA approach had managed to build up this level of diversity before the environmental change. The reason for the similar performance when initialized from the QDSA population could be due to a highly deceptive fitness landscape. This could mean that e.g. the EA on its own would be struggling to find a suitable morphology, whereas the seeded population of diverse morphologies may already contain a body in a good location of the search space, leaving time for controller optimization. The results from transitioning environments highlight two important aspects of diversity. Sufficient diversity is required to find solutions for difficult environments, and when experimenting with different environments in ER, it is difficult to a priori predict if a search algorithm is capable of finding a solution. However, search algorithms that produce and maintain diversity are more likely to handle the challenge.

The analysis of genealogical ancestry revealed several interesting aspects about how the population of solutions evolve for the three different search algorithms. Based on the number of ancestors and the age of individuals in the population (Figure 11), it can be seen that the generational replacement aspect of SOFO leads to many young solutions compared to the other approaches. This is to be expected and does not appear to disadvantage SOFO compared to MOFD in regard to performance. When looking at the difference between MOFD and QDSA it can be seen that QDSA is producing more solutions throughout evolution. One potential explanation for this is that due to the complex Pareto dominance calculated for solutions in MOFD solutions are rarely replaced based on fitness, and once diversity is maximized, as illustrated on the left in Figure 6, the search stagnates. This shows the complexities of introducing diversity into a maximization regime and it is likely that the two additional objectives are reducing the opportunity to improve on fitness.

Based on the QD metrics of the ancestry, shown in Figure 12, it can be seen that ancestors of solutions in QDSA cover a large area of the search space compared to the two other search algorithms. One could expect that since SOFO has many orders of magnitude more ancestors the solutions would cover a large area of the search space, however, the QD metrics show that the ancestors are not as morphological diverse as in QDSA. This result corroborates on the notion that MAP-Elites is better at generating *diverse* stepping stones, as proposed by Mouret and Clune (2015). This is also underscored by the ancestry’s QD-score (Figure 12), which shows that—in general, ancestors in QDSA are both diverse and high performing. Lastly, by building a linear model, predicting maximum fitness based only on coverage and QD-score of ancestry, we showed that diverse and high-performing ancestors could be a good predictor of performance. By modelling the relationship between genealogical ancestry and search performance, stepping stones can be seen in the larger context of evolution and gives an even stronger indication that MAP-Elites is able to produce impressive results based on diverse and high-performing intermediary solutions. By performing this analysis across populations, over many runs, we are able to gain statistical insight into the notion of stepping stones which strengthens the overall conclusion.

We showed how QDSA is an effective method for evolving both the morphology and control of modular robots for performance, diversification, and transfer to new environments. While the implementation of QDSA is promising for evolving both the morphology and control of modular robots, there are additional challenges related to how different selection methods could be further improved in QDSA. The modular robotics approach furthermore allows us to expand our module inventory to incorporate various types of other structural, sensing and actuator modules. This possible extension will convolute the search space further and possibly benefit more from QDSA than other algorithms. Experiments should also be conducted with different modular robotics systems to verify the results reported here for different modules, genotype-to-phenotype mappings, and tasks. One challenge with our setup is that different robotic blueprints can map to the same morphological features, which might be a problem as it can constrain the type of solutions found to particular robot morphologies. Therefore, additional morphological features could be implemented to create a multi-dimensional map that could lead to better and more unique solutions.

## 5 Conclusion

Optimizing both the morphology and controller for modular robots is challenging due to the large and unknown search space. As the amount of exploration vs. exploitation to be used for optimization strategies is usually determined by the complexity of the agent and the environment—the ruggedness of the resulting fitness landscape—we compared three evolutionary algorithms to determine how each performs on this challenging search space. The results showed that the QDSA approach based on MAP-Elites produced higher performing and more morphologically diverse solutions compared to the SOFO and MOFD approaches which were based on an objective-only EA and a diversity-augmented multi-objective EA, respectively. The importance of the diversity produced by MAP-Elites is corroborated through the successful transferal of evolved robots to two additional, and more difficult, terrain types. The genealogical ancestry produced by each algorithm furthermore indicated that MAP-Elites found more diverse and high performing stepping stones, shedding new light on how the algorithm achieves high-performing final solutions. The added pressure for diversification in the two morphological dimensions of the MAP-Elites-based approach bolsters how useful it can be for evolving both the morphology and control of modular robots.

## Data Availability

The datasets presented in this study can be found in online repositories. The names of the repository/repositories and accession number(s) can be found in the article/Supplementary Material.

## Footnotes

1 Source available at: https://github.com/nordmoen/gym-rem

2 For the complete analysis see the Supplementary Material at: https://folk.universitetetioslo.no/jorgehn/modular_journal/

## Appendix 1

**Table A1:** Morphological parameters for the evolved modular robots.

Parameter	Description	Value
*η*	Maximum module count	20
*δ*	Maximum module depth from root	4

**Table A2:** Parameters for the decentralized wave pattern controllers. The value ranges are based on the servo used in the real world modules.

Parameter	Description	Range
*θ*	Set-point angle	[−1.57,1.57]
*α*	Amplitude	[−1.57,1.57]
*ω*	Frequency	[0.2,2]
*ϕ*	Phase offset	[−2π,2π]
*o*	Amplitude offset	[−1.57,1.57]

**Table A3:** Values tested during meta-optimization of experiment parameters.

Parameter	Values
Probability of morphological mutation	[0.005,0.01,0.05,0.1,0.2,0.4]
Controller magnitude (*σ*)	[0.005,0.01,0.05,0.1,0.2]

**Table A4:** Coefficients for the model predicting fitness based on ancestry. The model has a residual standard error of 0.5441 with 599538 degrees of freedom.

Coefficient	Estimate	Standard error	*t*-value	P(>|t|)
$Coverage_{log}$	0.3241577	0.0004636	699.3	$<2e^{-16}$
QD-Score	0.0714622	0.0005870	121.7	$<2e^{-16}$
$Coverage_{log}$: QD-Score	−0.0127152	0.0001111	−114.4	$<2e^{-16}$
